# Separating Drought Effects from Roof Artifacts on Ecosystem Processes in a Grassland Drought Experiment

**DOI:** 10.1371/journal.pone.0070997

**Published:** 2013-08-01

**Authors:** Anja Vogel, Thomas Fester, Nico Eisenhauer, Michael Scherer-Lorenzen, Bernhard Schmid, Wolfgang W. Weisser, Alexandra Weigelt

**Affiliations:** 1 Institute of Ecology, Friedrich-Schiller-University Jena, Jena, Germany; 2 Department Umweltmikrobiologie, Helmholtz-Zentrum für Umweltforschung – UFZ, Leipzig, Germany; 3 Faculty of Biology, Geobotany, University of Freiburg, Freiburg, Germany; 4 Institute of Evolutionary Biology and Environmental Studies, University of Zurich, Zurich, Switzerland; 5 Terrestrial Ecology Research Group, Department of Ecology and Ecosystem Management, Center for Food and Life Sciences Weihenstephan, Technische Universität München, Freising, Germany; 6 Institute of Biology, University of Leipzig, Leipzig, Germany; USDA-ARS, United States of America

## Abstract

**1:**

Given the predictions of increased drought probabilities under various climate change scenarios, there have been numerous experimental field studies simulating drought using transparent roofs in different ecosystems and regions. Such roofs may, however, have unknown side effects, called artifacts, on the measured variables potentially confounding the experimental results. A roofed control allows the quantification of potential artifacts, which is lacking in most experiments.

**2:**

We conducted a drought experiment in experimental grasslands to study artifacts of transparent roofs and the resulting effects of artifacts on ecosystems relative to drought on three response variables (aboveground biomass, litter decomposition and plant metabolite profiles). We established three drought treatments, using (1) transparent roofs to exclude rainfall, (2) an unroofed control treatment receiving natural rainfall and (3) a roofed control, nested in the drought treatment but with rain water reapplied according to ambient conditions.

**3:**

Roofs had a slight impact on air (+0.14°C during night) and soil temperatures (−0.45°C on warm days, +0.25°C on cold nights), while photosynthetically active radiation was decreased significantly (−16%). Aboveground plant community biomass was reduced in the drought treatment (−41%), but there was no significant difference between the roofed and unroofed control, i.e., there were no measurable roof artifact effects.

**4:**

Compared to the unroofed control, litter decomposition was decreased significantly both in the drought treatment (−26%) and in the roofed control treatment (−18%), suggesting artifact effects of the transparent roofs. Moreover, aboveground metabolite profiles in the model plant species *Medicago x varia* were different from the unroofed control in both the drought and roofed control treatments, and roof artifact effects were of comparable magnitude as drought effects.

**5:**

Our results stress the need for roofed control treatments when using transparent roofs for studying drought effects, because roofs can cause significant side effects.

## Introduction

Precipitation change is an important driver of global change affecting the functioning of ecosystems [1] and is predicted to increase in the future [2]. Consequently, many experiments have investigated the effects of precipitation change on ecosystem functioning. However, the experimentally applied setup of drought experiments may have side effects, hereafter called artifacts, in addition to the intended manipulation of precipitation patterns (summarized in [3]). The implications of such artifacts are particularly serious, when a second important driver of global environmental change with effects on ecosystem functioning, like plant diversity loss (e.g., [1], [4]), is manipulated, as it is not known how potential artifacts would interact with a second treatment. Given the importance of multifactor experiments for quantifying the effects of global change on ecosystem functioning, it is crucial to critically evaluate the results and potential conclusions drawn from drought experiments.

Roofs or rain-out shelters are a common tool to induce drought in field experiments. Their design varies among experiments, e.g. they vary in size, shape and transparent material [5]. All roofs are constructed in a way to minimize possible artifacts, which is difficult and rarely tested. The most obvious unwanted artifacts are shading (e.g., [6], [7]), and passive warming [8], [9], although some authors report only slight increases in air and soil temperature due to roofs [6], [10], [11] or even no influence [12], [13]. In contrast to experiments in forests, where rain can be intercepted below the canopy, roofs in grassland studies must cover the entire plant stand, and thus artifacts might be particularly significant in these ecosystems.

Warming and changes in irradiance may be part of the predicted climate change in certain scenarios [14], [15], and one could argue that such roof effects might help to simulate future climate more realistically. Nevertheless, artifacts are not controlled in roof experiments and may not mirror regional projections [2], [15]. For example, with increasing frequency of heat waves and drought periods, irradiance is more likely to increase than decrease. Furthermore, roof artifacts may themselves affect ecosystems and therefore confound the results of drought experiments. Warming for example has an effect on several ecosystem functions and increases productivity and decomposition [16], [17], [18]. Both, drought and roof artifacts might differ in various aspects, as, e.g., different plant communities, and therefore confound the results on interactions.

Shifts in plant diversity are another important driver of global environmental change in addition to climate change. And as both drivers operate simultaneously in real ecosystems, their interacting effects are of particular importance for future studies. However, plant diversity changes the density and aboveground productivity of the plant communities and therefore community structure. Thus, if the interaction of drought with another treatment (i.e. plant diversity) is investigated, one should be aware of the interacting effects of roof artifacts with this second treatment to avoid misinterpretations. For example, species richness was found to decrease resistance of biomass production to drought ([19], [20], but see [21]), but it is unknown, so far, whether this relationship was partly confounded by roof artifacts.

As there is currently no way to prevent unwanted roof effects, additional control treatments are needed to separate drought effects from roof artifacts. An obvious control involves roofs under which the collected rain is redistributed to the experimental plots [13], [22], [23], named “roofed control” hereafter. To our knowledge, there is no published study, investigating whether roof artifacts affect ecosystem processes and therefore confound the conclusions of drought experiments. Furthermore, studies using roofed controls in highly replicated experiments manipulating a second treatment, such as plant diversity experiments, do not exist.

We performed a roof experiment to separate pure drought and roof artifact effects and their influence on three ecosystem functions, aboveground biomass production, litter decomposition and the production of plant metabolites. This experiment was part of a large grassland biodiversity experiment and therefore allowed us to study, whether the effects of roof artifacts varied with a second treatment causing confounding interaction effects between drought and the second treatment. We compared measurements from roofed and unroofed control treatments to assess roof artifacts and to quantify their relative strength compared to the drought effects themselves. We tested 1) whether the roofs caused potential artifacts such as shading or passive warming, 2) the extent of artifact effects on aboveground biomass production, litter decomposition and the production of plant metabolites compared to drought and 3) whether any of these effects interacted with a second treatment, i.e. plant diversity.

## Methods

### Experimental Design and Drought Manipulations

The Jena Experiment field site in the floodplain of the river Saale in Jena (Thuringia, Germany, 50°55′N, 11°35′E, 130 m a.s.l.) served as platform for our experiment. In 2002, 80 plant communities (experimental plots) were established and assembled out of a pool of 60 mesophilic grassland species typical for the regional Molinio-Arrhenateretea meadows. The plots were arranged in four blocks perpendicular to a gradient in soil texture and moisture from the river Saale. The plant communities varied in species richness (1, 2, 4, 8, 16 and 60 species) and functional group richness (1, 2, 3 and 4 functional groups: grasses, small herbs, tall herbs, legumes). This experimental design was maintained by two to three annual weeding campaigns to eliminate non-target plants. The field site was managed by mowing twice a year (beginning of June and beginning of September). For details of the experimental design see Roscher et al. [24].

**Figure 1:**
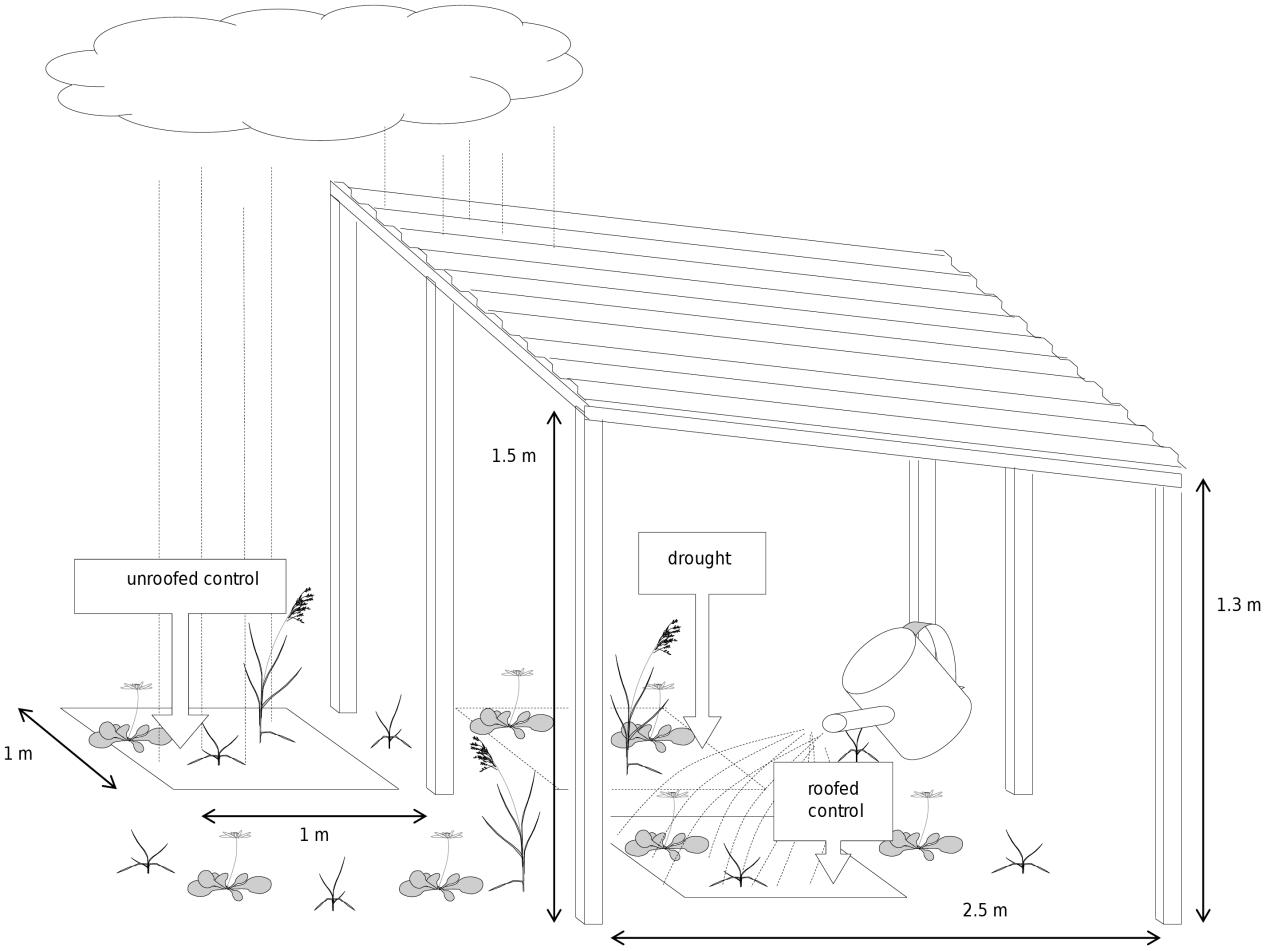
Roof construction and arrangement of the subplots in the field site. Given are the different subplots and the size of subplots and the roof construction. For more details see main text.

Since summer droughts are forecasted to increase in the future [2], our roof treatments excluded all rainfall from July through August every year since 2008. Starting in 2009 (16-Jul to 01-Sep-2009 and from 25-Jul to 03-Sep-2010 and from 11-Jul to 29-Aug-2011), we changed the roof constructions and applied three roof treatments to each of the 80 plots (which means 80 replicates per roof treatment): a roofed treatment without water addition served as “**drought**” treatment, a roofed treatment with water addition served as “**roofed control**” treatment and an ambient treatment served as “**unroofed control**” (Fig. 1). The roof, which covered “drought” and “roofed control” subplots, consisted of a wooden frame of 3×2.5 m and was covered with corrugated PVC sheets of 1.0 mm thickness (www.paruschke-kunststoffe.de, product code: PVCSPK7018K10). It had a height of 1.3 m to 1.5 m for good ventilation with a roof inclination of 4.6° for precipitation runoff. The large size of the roofs enabled us to let the removed water drop on the nearby ground, because we could perform our measurements in an area (1×1 m^2^), which was 1 m apart from the lowest edge and about 0.5 m from the other edges of the roofs to minimize edge effects. With this design, the shelters prevented direct precipitation on the subplots, when rainfall was straight or slightly skewed and water only entered on very windy days. However, lateral movement of water by overland flow or lateral diffusion in the soil was possible since the plots were not trenched and water was not collected. Given that the field site is located in a plane area, we expect overland water flow to only occur during extreme rain events. For watering the “roofed control” we collected rain water of a small subset of roofs equipped with guttering and plastic barrels. To prevent the development of algae and to mimic the ambient precipitation pattern as closely as possible, we always watered the subplots the day after each rain event. We added the amounts of precipitation to the “roofed control”, which were recorded in 10-minutes intervals at a weather station located on the field site apart from the experimental plots.

### Data Sampling

We used a subset of plots for the measurements of potential artifacts and soil moisture to be able to record data simultaneously across a given time interval. We measured potential roof artifacts concerning shading and passive warming as well as litter decomposition in the first year of the experimental manipulations (2009). Soil moisture and biomass data were collected in every year. Measurements of air and soil temperatures were carried out in four plots, one in each block, to cover the edaphic variability across the field site, which means four replicates per roof treatment. The selected plots had similar species richness and high grass cover (two plots with one and one plot with two grass species, one plot with a grass and a small herb species). Soil moisture was measured as volumetric water content every 15 minutes by EC-5 sensors and recorded with Em50 data loggers (Decagon devices, Pullman, USA) continuously since 12-May-2009 in about 7 cm depth of drought, roofed and unroofed control subplots of three of the above mentioned plots, which means three replicates per roof treatment. Soil temperature (in 7 cm depth) and air temperature (in 25 cm height) was recorded by testostor 175 data logger (Testo AG, Lenzkirch, Germany) every 15 minutes between 12-May and 05-Nov-2009.

PAR was measured above the vegetation and in a lower height compared to the roofs in all subplots of four plots, giving again four replicates per roof treatment. In contrast to the selected plots for soil parameters described above, these plots were close to each other for practical reasons. We recorded PAR every 30 minutes from sunrise to sunset on 19-Aug-2009 using the portable sunscan system SS1 (Delta-T, Cambridge, UK).

Aboveground biomass and litter decomposition was measured in all subplots of all 80 plots. The aboveground plant material was cut at a height of 3 cm above soil surface within one frame of 20×50 cm per subplot at the end of the drought periods in 2009 and 2010 (28- to 31-Aug-2009 and 25- to 26-Aug-2010). Plant material was sorted into sown species, unsown species (weeds) and dead material, dried (70°C, 48 h) and weighed separately. Aboveground biomass presented here represents standing biomass (dry mass) of sown species.

Litter decomposition was measured using plastic containers (9×9 cm in size). which were constructed using pots with 4 mm mesh at the bottom. Mesh and lateral cuttings of the pots allowed access of micro-, meso- and macrofauna to litter material. We used ∼3 g of dried senescent wheat shoot material (chopped into pieces of ∼3 cm, N = 0.4%, C = 45.2%, C:N ratio = 111.5) as standard litter in one container per subplot from 17-Jun to 24-Aug-2009. At the end of the experiment, containers were collected, and the remaining litter material was dried (70°C, 48 h) and weighed.

For the analysis of metabolites, we used the legume *Medicago x varia*. Flower buds (sampled from the apical nodes of leading shoots and showing no signs of petal pigmentation), sink leaves (sampled from the apical nodes of leading shoots) and source leaves (sampled from the 3^rd^ or 4^th^ node below the apical meristem from leading shoots) were harvested from identical plant individuals. We harvested plant material (organs from one plant per subplot) in the fourth year of consecutive summer drought on 22-Aug-2011 from 11 a.m. to 3 p.m. in every subplot of eight plots. In addition, we sampled sink leaves and source leaves from at least seven different individuals from each subplot of a plot of high species cover, to get an estimate of the within-subplot variation. Samples were weighed, frozen immediately in liquid nitrogen and stored at –80°C. Metabolite extraction, derivatization and gas chromatography were done as described in Fester *et al.* [25] based on methods described by Sanchez *et al.* [26] and Desbrosses *et al.* [27]. In short, the frozen material was extracted with methanol and chloroform with the addition of ribitol (0.2 mg/ml dissolved in methanol) as internal standard. Derivatization involved treatments with methoxamin hydrochloride (20 mg/ml in pyridine) and *N*-methyl-*N*-(trimethylsilyl)-trifluoroacetamide. Samples of 1 µl were analyzed in splitless mode using an Agilent GC 6890 equipped with a Rtx-5Sil MS capillary column (30 m×0.25 mm inner diameter; Restek GmbH, Bad Homburg, Germany) and a MSD 5973 (Agilent, Böblingen, Germany). Data evaluation was performed using the programs Metalign [28] for baseline correction and TagFinder [29] for chromatographic deconvolution and quantification of compounds. Retention time indices (RI) calculated by TagFinder and mass fragmentation data were compared with data from the Golm metabolome database [30], [31] for metabolite identification.

### Statistical Data Analysis

Data on soil moisture, temperature, PAR, aboveground biomass and litter decomposition were analyzed with linear mixed models using the REML algorithm to estimate variance components; and F statistics to test fixed effects. Depending on the dataset and the underlying experimental setup, different sets of fixed and random terms had to be used for different response variables (Table S1). For most analyses the treatment term was split into two contrasts. The first contrast distinguished between either roofed (mean of drought and roofed control treatment) and unroofed or between “moist” (mean of roofed and unroofed controls) and “dry” (drought) treatments. As a second contrast we tested for the **pure drought** effect and compared the (roofed) drought treatment and the roofed control, or we tested for **roof artifacts** and compared the roofed and unroofed control. Temperature was analyzed independently for day and night with the official time of sunrise and sunset as separator. To test whether temperature changed due to roof effects or by local climate, we fitted a contrast, which distinguished between cold and warm days (air: ≥24°C; soil ≥20°C) or cold and warm nights (air: ≥15°C; soil ≥19°C) using data from the field weather station. For PAR we fitted two models, one with all data and one with only noon data (11 am –3 pm) to separate whole day effects from effects of high radiation conditions. Models of time series data (soil moisture, air and soil temperature) were fit using an autoregressive correlation structure of order one (AR1) for the residuals to account for serial correlations. In the case of PAR this model adjustment was dispensable due to a sinus contrast for time which describes the sinus shaped curve of PAR over the day. For aboveground biomass and litter decomposition we fitted several models and included species richness (log-linear), functional group richness, the presence of single functional groups, the treatment contrasts and the two-way interactions of the diversity variables with the treatment contrasts in the fixed-terms part of the model. Because functional group richness and the presence of single functional groups were rarely significant, we excluded them from the models. Mixed effects models were performed using GenStat Release 15.1 (VSN International Ltd.).

Multivariate analysis of metabolite levels of all three plant organs in combination (data from source leaves, sink leaves and flowers) was performed by partial least square discriminant analysis (PLS-DA) using the function plsda() from the R-package *caret* [32] in R 2.14.0 [33]. PLS-DA accounts for the group structure (in our case the roof treatments) of the dataset while still being capable for dimension reduction [34], [35] and tests how well the treatments can be separated. We did not use unsupervised methods which can identify the gross variability in a multivariate dataset, because we were not interested in other factors, which might also determine metabolite levels and cannot be tested due to the low numbers of replicates (e.g. species richness or community composition). Metabolite data were fitted to PLS-DAs using the function envfit() from the R-package *vegan* [36]. This function assessed significance of correlations by a correlation test using Monte Carlo permutations (N = 999) of the fitted vectors [37]. The goodness of fit statistics used was squared correlation coefficient (Pearson’s product moment correlation coefficient). Significant differences in individual metabolites were analyzed with the same mixed model approach (Table S1) as described for the ecosystem processes excluding, however, the species richness term due to the low numbers of replicates. Data were log- (soil moisture, biomass, metabolites) or square root-transformed (PAR) in case residuals were not normally distributed.

## Results

### Soil Moisture

**Figure 2:**
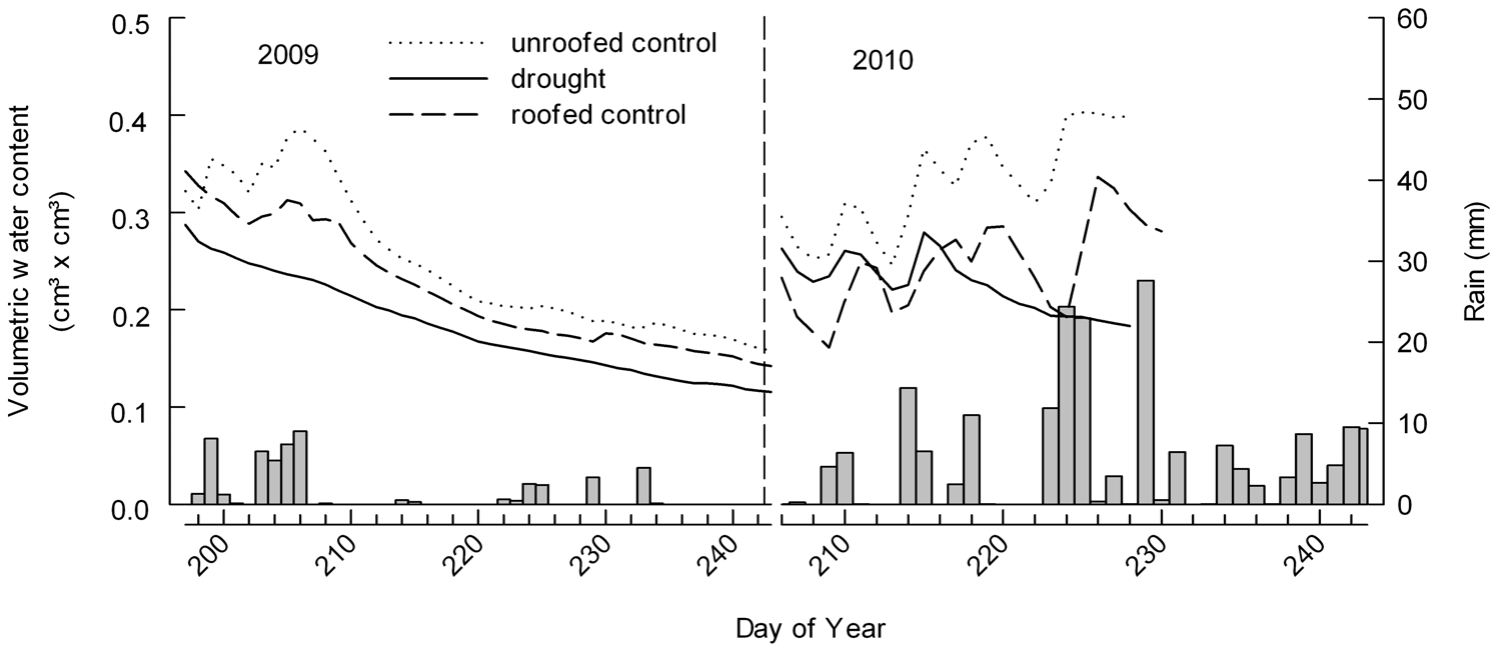
Soil moisture and daily precipitation patterns during the period of induced drought. In summer of 2009 (left) and 2010 (right). Soil moisture data are shown for all three roof treatments (lines, average of N = 3 plots). Daily precipitation patterns (grey bars) were measured on the field site of the Jena Experiment.

In 2009, 53.7 mm rainfall was excluded during the drought period. and precipitation patterns approximated the long-term seasonal trend with the exception of an unusually dry winter (January to March) and a wet autumn (October to December, Table 1). During spring and summer (April to September), April and July were wetter whereas June and especially August were dryer than the long-term average. In 2010 we observed a higher annual precipitation compared to the long-term mean and a higher intra-annual variability (Table 1). As in 2009, winter was dryer and autumn wetter compared to the long-term mean. The high precipitation occurred during summer (August), whereas spring (April and June) was dry. During the drought period in 2010, 196.7 mm rainfall was excluded. In 2009 soil moisture did not differ significantly between the treatments (F_1,6.1_ = 0.77, p = 0.414) but we found a significant reduction of soil moisture in response to drought over time in 2010 (F_1,5.1_ = 186.79, p<0.001), whereas soil moisture under ambient conditions and in the roofed control treatment increased over time (Fig. 2).

**Table 1:** Climatic parameters measured on the field site of the Jena Experiment during the study years 2009 and 2010 with the reference period 1961–1990 measured by the German Weather Service DWD in the city center of Jena.

	Air temperature (°C)			Precipitation (mm)			Soil moisture (Vol%)		Soil temperature (°C)	
	1961–90	2009	2010	1961–90	2009	2010	2009	2010	2009	2010
**January**	0.40	−3.09	−3.92	37.0	9.0	11.0	22.11	35.56	−0.20	1.02
**February**	1.40	1.15	0.12	34.0	33.7	23.6	33.31	35.26	1.34	0.61
**March**	4.80	5.04	4.89	43.0	42.5	29.1	37.01	36.65	5.25	4.82
**April**	8.60	11.58	8.74	57.0	73.7	19.8	31.39	33.04	12.13	9.39
**May**	13.40	13.89	11.13	62.0	62.6	93.0	31.05	31.49	15.37	12.43
**June**	16.70	15.01	16.89	75.0	52.9	20.4	28.58	26.70	16.50	17.62
**July**	18.20	18.34	20.66	52.0	85.1	88.6	31.29	22.95	19.75	21.91
**August**	17.40	18.59	16.76	63.0	14.6	184.2	22.34	33.15	19.13	18.36
**September**	14.20	14.56	12.67	42.0	53.6	64.7	23.67	29.98	15.85	14.15
**October**	9.80	8.42	8.26	39.0	47.3	19.4	28.70	30.91	10.31	9.73
**November**	5.00	8.06	5.59	41.0	68.3	94.5	34.00	33.95	7.57	7.28
**December**	1.70	0.64	−4.12	42.0	80.0	56.3	36.22	35.88	3.19	2.20
**Year**	**9.30**	9.35	8.14	**587.0**	623.3	704.5	29.97	32.13	10.52	9.96

### Air and Soil Temperature

**Figure 3:**
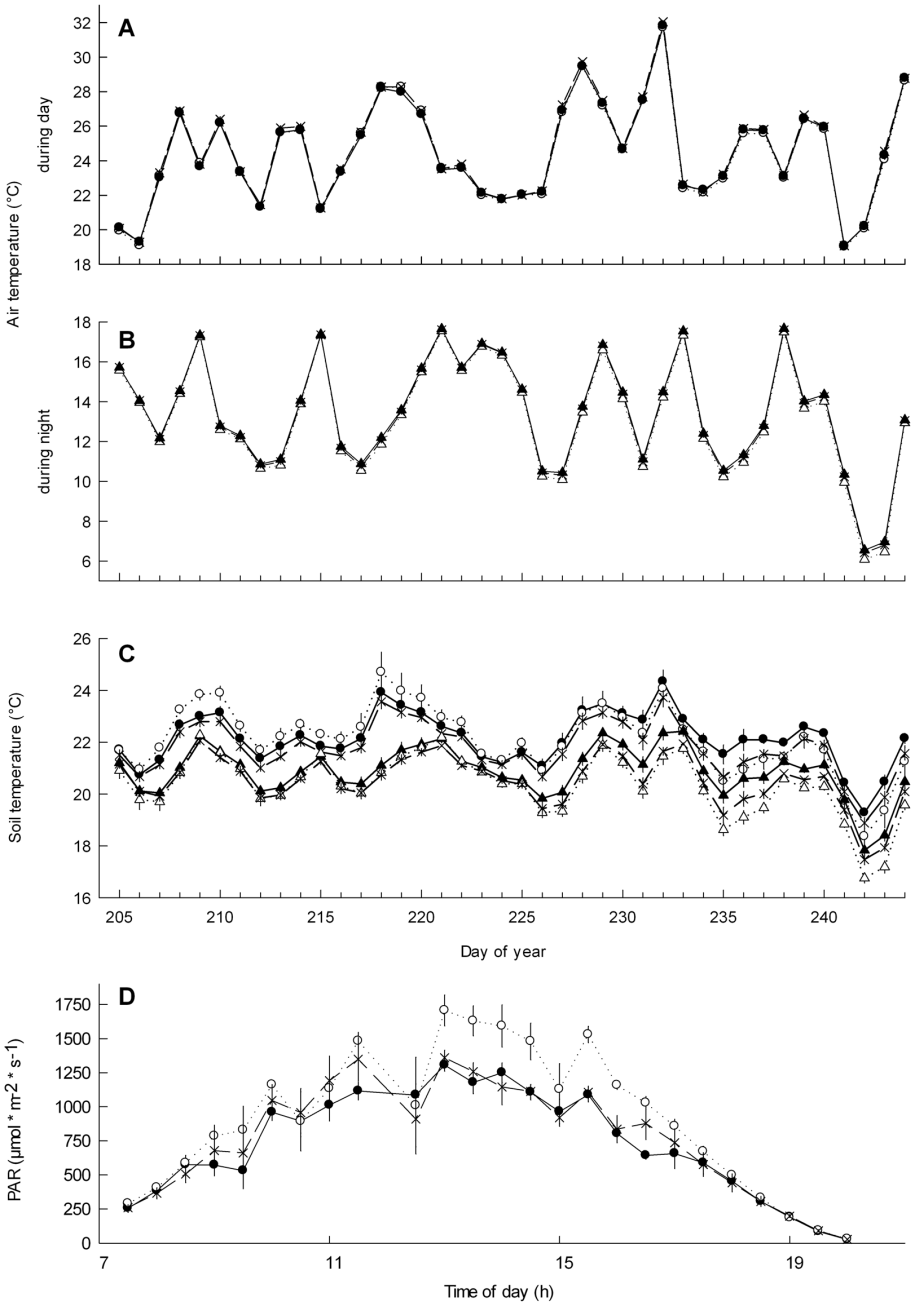
Effects of the presence of roofs on abiotic parameters: air temperature (a, b), soil temperature (c) during day (circles) and night (triangles) and photosynthetically active radiation (d). Given are means and standard errors of the drought treatment (filled symbols, solid lines), unroofed (open symbols, short dashed line) and roofed controls (x symbols, long dashed line) for day (circles) and night (triangles). Data represent mean and standard error of all three treatments in four (respectively three in case of temperature) plots.

Air temperature during the day was not significantly affected by the roofs (Fig. 3A). In contrast, air temperature during the night was significantly increased by 0.14°C due to the roofs (F_1,6_ = 32.96, p<0.001, Fig. 3A, B), irrespective of whether nights were cold or warm. Soil temperature during the day was significantly decreased by ∼0.45°C under roofs in comparison to ambient, but only on warm days (significant two-way interaction of roofed/unroofed-contrast × cold/warm contrast: F_1,5.7_ = 15.89, p<0.01; Fig. 3C). In contrast, soil temperature during the night was increased by the roofs by 0.25°C, but only on cold nights (significant two-way interaction of roofed/unroofed-contrast × cold/hot contrast: F_1,5.1_ = 25.6, p<0.01).

### Photosynthetically Active Radiation (PAR)

The roof (F_1,9_ = 19.23, p = 0.002) and time of day (F_1,25.5_ = 200.49, p<0.001) showed significant effects on PAR (Fig. 3D). Roofs reduced PAR by around 15% (corrected mean over the whole day), and by around 16% during noon (F_1,9_ = 5.39, p<0.045).

### Aboveground Biomass

**Figure 4:**
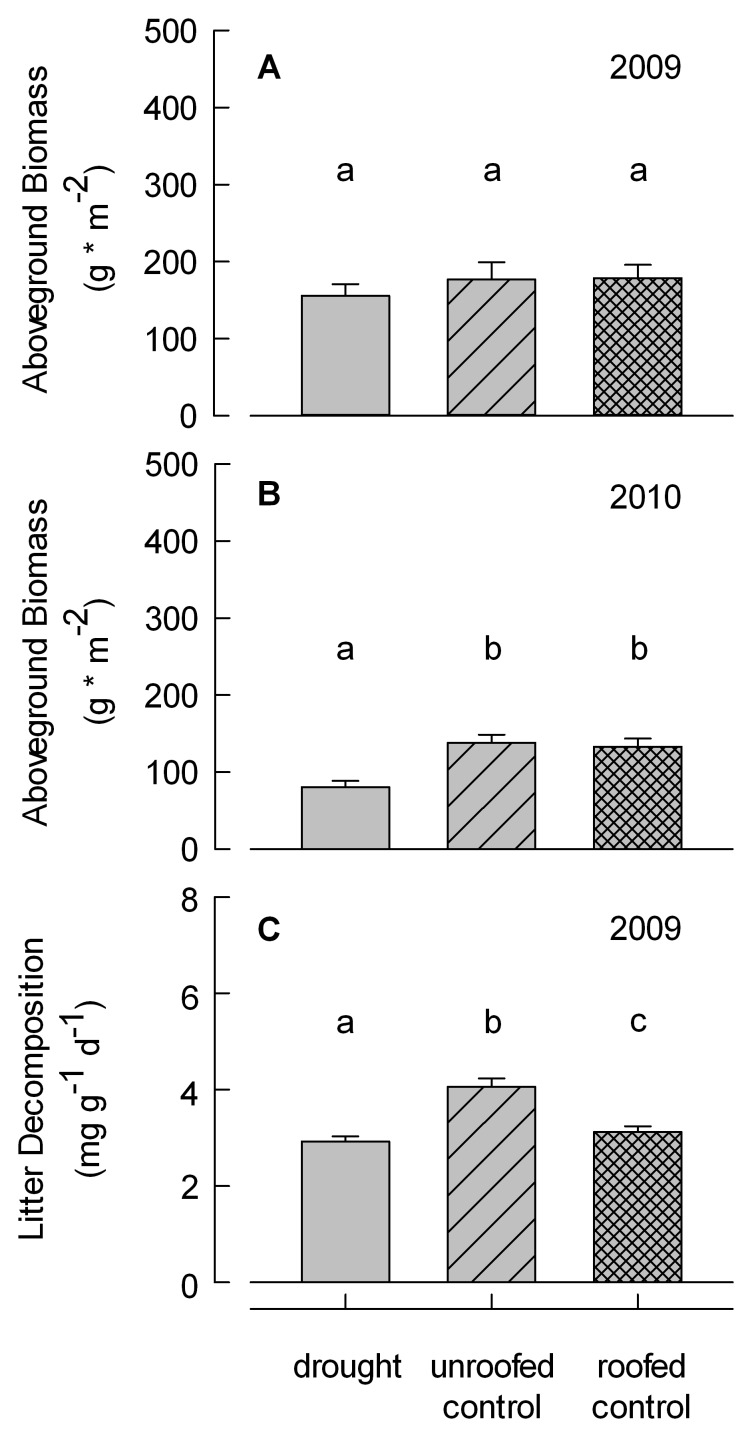
Treatment effects on ecosystem properties. Aboveground biomass production (a, measured in 2009 and b, measured in 2010) and litter decomposition (c). Data represent mean and standard error of all three treatments in 76 plots.

Aboveground biomass after drought was significantly affected by the treatments only in 2010, but not in 2009 (Table 2, Fig. 4A and B). In 2010 mean biomass was similar in unroofed (mean ± standard error: 132.0±10.5 g * m^−2^) and roofed control plots (129.9±10.7 g * m^−2^) and significantly lower in drought plots (76.9±8.1 g * m^−2^). Although biomass was not significantly different between roof treatments in 2009, the pattern was the same (ambient: 168.4±23.2 g * m^−2^; roofed control: 170.8±17.5 g * m^−2^; drought: 151.3±15.5 g * m^−2^). We did not find a significant interaction between species richness and roof artifacts in either year (Table 2). The relationship between plant diversity and biomass was positive in all treatments.

**Table 2:** Summary of mixed effects models for aboveground biomass in August 2009 and 2010 as well as for decomposed wheat litter.

		Biomass 2009			Biomass 2010			Decomposition 2009		
	df_Num_	df_Den_	F	*p*	df_Den_	F	*p*	df_Den_	F	*p*
**Model A**										
Species richness (log-scale) = SR	1	69.2	27.9	<0.001***	70.8	21.6	<0.001***	71.3	20.0	<0.001***
Presence of Legumes	1	67.7	8.2	0.006**						
Treatment										
roofed vs. unroofed = DRvsU	1	141.2	1.6	0.205	140.8	6.3	0.014*	146.8	59.8	<0.001***
DvsR	1	141.0	1.8	0.188	141.6	33.3	<0.001***	146.8	4.2	0.042*
Treatment × SR										
DRvsU × SR	1	143.4	2.9	0.092	141.4	0.6	0.453	146.5	2.3	0.131
DvsR × SR	1	143.0	0.4	0.513	144.0	0.7	0.404	147.4	0.7	0.414
**Model B**										
Species richness (log-scale) = SR	1	69.2	27.9	<0.001***	70.8	21.6	<0.001***	71.3	20.0	<0.001***
Presence of Legumes	1	67.7	8.2	0.006**						
Treatment										
dry vs. wet = DvsRU	1	141.2	0.3	0.611	141.3	39.0	<0.001***	146.6	32.0	<0.001***
RvsU	1	140.6	3.1	0.08	140.8	0.6	0.453	147.3	32.0	<0.001***
Treatment × SR										
DvsRU × SR	1	143.6	0.1	0.787	142.9	1.2	0.271	146.7	0.0	0.948
RvsU × SR	1	141.8	3.2	0.075	142.0	0.04	0.834	147.7	3.0	0.087

For the analysis we used all plots of the Jena experiment, except for the 60-species mixtures. In Model A the treatment contrast was split into a contrast for roofed versus unroofed subplots (DRvsU), which aggregates the drought (D) and roofed control (R) treatments and tested it against the unroofed (U) treatment, and the residual contrast (DvsR), which considers the pure drought effect. In Model B the treatment contrast was split in a contrast of dry versus wet subplots (DvsRU), which aggregates the unroofed and roofed control treatments and tested it against the drought treatment, and the residual contrast (RvsU), which considers the roof artifact effects. Df = degrees of freedom; Num = numerator; Den = Denominator.

### Litter Decomposition

Litter decomposition was affected by the roof construction (Table 2, Fig. 4C). Litter decomposition was highest in the unroofed control (3.9±0.1 mg * g^−1^ * d^−1^), moderate in the roofed control treatment (3.2±0.1 mg * g^−1^ * d^−1^) and lowest in the drought treatment (2.9±0.1 mg * g^−1^ * d^−1^). The pure drought effect was small compared to the roof artifact (Table 2, Fig. 4C). There was no interaction between species richness and the pure drought or roof artifact (Table 2).

### Metabolites

**Figure 5:**
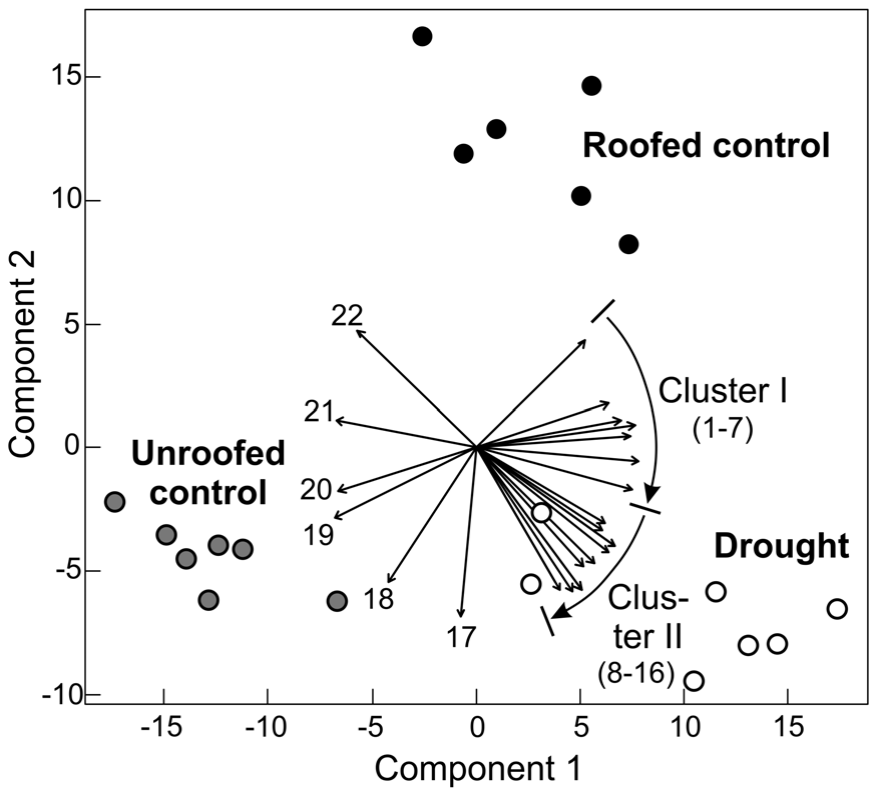
Partial least square discriminant analysis (PLS-DA) of metabolite profiles. (combined data of flowers, sink leaves and source leaves) with one individual (*Medicago x varia*) analyzed for each subplot out of seven plots. Metabolites correlating significantly with PLS-DA data (according to Monte Carlo-permutations, p<0.05) are represented as black arrows. Only identified metabolites are shown. These metabolites comprise: asparagine/flower (1), arabitol/flower (2), arabitol/sink leaf (3), citric acid/sink leaf (4), allantoin/flower (5), pinitol/sink leaf (6), asparagine/source leaf (7), arabitol/source leaf (8), 1,6-anhydro-glucose/source leaf (9), sorbitol/source leaf (10), 2,4-diamino-butanoic acid/source leaf (11), glucose/source leaf (12), erythritol/source leaf (13), beta-alanine/sink leaf (14), xylitol/source leaf (15), kestose/sink leaf (16), galactinol/flower (17), mannose/sink leaf (18), glucose-6-phosphate/sink leaf (19), phenylalanine/source leaf (20), phosphoric acid monomethyl ester/sink leaf (21), threonine/sink leaf (22). Retention time index and fragmentation pattern were not sufficient to differentiate between closely related isomers in the case of arabitol, xylitol, 1,6-anhydro-glucose, sorbitol, kestose, galactinol and mannose.

In total, 227 different analytes could be detected in each plant organ, of which 34% could be specified. Multivariate analysis (PLS-DA) of the metabolite profiles from all organs per plot and subplot clearly separated the different roof treatments (Fig. 5). PLS-DA-component 1 separated between the roofed (drought, roofed control) and unroofed treatments. PLS-DA-component 2 separated between the “moist” (roofed and unroofed controls) and the drought treatment. PLS-DA of sink leaves and source leaves obtained from plants from one single plot gave similar results (data not shown). We observed 66 analytes with significant correlations (Monte Carlo-permutations, p<0.05), when correlating data of individual metabolites with PLS-DA-components. 22 of these analytes were identified and included in Fig. 5. A subset of these 22 analytes can be grouped into two clusters (cluster I and II), which both positively correlate with the roof effect (component 1). These clusters primarily comprise nitrogen-containing compounds, sugars and sugar alcohols, with nitrogen-containing compounds being predominant in cluster I and sugar or sugar alcohols being predominant in cluster II. Interestingly cluster II almost exclusively comprised compounds from source leaves while cluster I comprised mainly compounds from all sink leaves and flowers. There was no drought effect on cluster I (no correlations with component 2), while we found a positive correlation between drought and cluster II. Analysis of significant differences in the levels of individual metabolites revealed drought effects on 20 analytes and effects of the roof artifacts on 27 analytes in at least one plant organ (Table S2). Only three of these analytes were affected both by pure drought and roof artifacts.

## Discussion

Many methodological issues are prevalent with drought studies in the field. The problem of comparison arises from the different experimental designs and the size of roof constructions, from missing common metrics to describe the magnitude of the treatment and from the question, how to deal with roof artifacts [3], [5], [38], [39]. The goal of the present study was to investigate the magnitude and ecological relevance of confounding roof artifacts in a drought experiment and their interaction with a second treatment, plant diversity. We found minor effects of passive warming (i.e. soil and air temperature were warmer at night and soil temperature cooler during the day) and strong shading effects of the roofs, confounding both, litter decomposition and plant metabolite profiles, while aboveground biomass remained unaffected. Moreover, roof artifacts did not interact with plant diversity as an additional experimental treatment.

Our roof construction induced a slight increase of air temperature during the night, indicating that roofs retarded cooling by blocking the emission of thermal energy from the soil surface. However, this did not lead to a significant passive warming effect during the day and indicates good ventilation of the roof constructions. Soil temperature differences between roofed and unroofed subplots were low compared to other experiments ([8], [22], [40]. Concerning shading, our measurements support previous results (e.g. [6], [22]) showing that fixed roofs reduce radiation. Automatically closing constructions might minimize or even prevent such roof artifacts, because they only close during rain events and slide back in dry periods [41], [42]; although this approach is appropriate, it mostly is not affordable, particularly when having a large number of roof replicates. Similarly, reducing precipitation with portable rain-out shelters is very laborious and only possible when working with a low number of replicates [43].

The ecological relevance of roof artifacts strongly depended on the ecosystem process. Despite the fact that light quantity and temperature have an influence on photosynthesis [44], [45] and hence growth, plant community aboveground biomass was not significantly different between the unroofed and the roofed control, indicating roof artifact effects to be small and independent of differences in the plant communities (as roof artifacts did not interact significantly with our second treatment). Drought itself did have the expected strong effect on aboveground biomass production, which highlights the strong dependency of plant biomass production on soil water availability.

For surface litter decomposition, however, we found evidence for significant roof artifact effects. The presence of a roof had stronger effects on litter decomposition than the induced drought, and both effects operated in the same direction by decreasing decomposition rates. Therefore, roof artifacts turned out to be very important for surface litter decomposition, and comparing a drought treatment using a roof with an unroofed control only, would most likely lead to a strong overestimation of drought effects on decomposition. The confounding roof effects on litter decomposition also did not vary across the second treatment, indicating similar confounding effects irrespective of the plant communities. We can only speculate why the roof construction reduced litter decomposition in such a strong way. Austin & Vivanco [46] found a strong impact of photodegradation on litter decomposition in a semi-arid ecosystem. If radiation was completely blocked, decomposition was reduced by 60%, if only UV-B radiation was blocked, there was already a reduction of decomposition by 33%. Photodegradation, especially degradation due to UV radiation, may play a particularly prominent role in arid and semi-arid ecosystems (e.g., [47]), but there is evidence that this mechanism is also important for a wider range of grasslands, including more mesic sites [48], [49]. Our experiment was not designed to determine the mechanism behind these effects, but assuming photodegradation plays a significant role in our system, it is conceivable that decomposition was sensitive to the roof artifacts through changes in radiation. Since we have not measured the spectral characteristics of solar radiation below the roofs, we cannot separate the effects originating from changes in light quantity or quality. However, we speculate that changes in light quality, with some blocking of UV radiation, might have had strong effects. Temperature effects of the roof construction seem not to be relevant for the decrease of decomposition under the roofed compared to the unroofed control, because increasing air temperatures should result in increasing decomposition rates when soil moisture is not limiting [17], [50] and therefore should act in an opposite direction compared to our results. Still, drought is also known as a factor that decreases litter decomposition, as demonstrated in laboratory experiments [50], [51] or in field experiments with automatically closing rain shelters [52], which do not have strong shading effects. Nevertheless, drought effects on litter decomposition might be largely overestimated, if roof artifacts are not taken into account [53], [54]. As litter decomposition is a key process for the global carbon cycle, it is crucial to separate climatic effects from methodic artifacts. However, little is known about the global role of photodegradation and about the influence of different roof types on this process.

Metabolite profiling of above-ground organs from *Medicago x varia* was chosen as a test for more subtle physiological changes induced by roofs, since there had been indications from another rain exclusion experiment that roofs may impact plant metabolite profiles independently from water availability (data unpublished). In accordance with this prior experiment, the clear distinction of metabolite profiles of the roofed control plants in comparison to all other treatments indicates roof artifact effects at the plant physiological level. While analysis using linear mixed models only resulted in a low number of identified significant changes in metabolite levels in our experiment, analysis of metabolites correlating with PLS-DA components showed higher numbers of significant correlations. The large numbers of sugars and sugar alcohols from cluster I and II in Fig. 5 are of particular interest in our context, since they represent compatible solutes typically accumulating under conditions of abiotic stress [55]. It is interesting to note that cluster II, which correlated with both roof and drought effects, comprised compounds almost exclusively from source leaves. Cluster I, in contrast, correlated exclusively with roof effects, comprised compatible solutes from flowers or sink leaves as well as some nitrogen containing compounds. While these observations are difficult to interpret in detail, they nevertheless allow the conclusion that, independently from drought levels, roofs introduced some kind of stress to the plant and that the impact of this stress was stronger than the impact of drought under the conditions prevailing in our experiment. As we did not find confounding roof artifacts on aboveground plant community biomass, the physiological changes induced by the roofs apparently were not strong enough to result in a significant change at the level of plant community biomass. However, they should be taken as a caution that other ecosystem processes that are more sensitive to changes in plant physiology may well be affected, as, e.g., the resistance of plants against herbivores. In addition, our experiment was short-term.We cannot exclude the possibility that persistent maintenance of drought treatments for more than three years might result in artifacts in plant physiological traits and total biomass production.

## Conclusions

In field drought experiments it is important to critically test for confounding roof artifacts to be able to correctly predict the consequences of drought. Our analysis shows that, in contrast to aboveground biomass production, roofs had clear confounding effects on litter decomposition and also affected plant physiology. We therefore strongly recommend the use of roofed controls for long-term and short-term studies. As the confounding roof artifacts did not interact with a second treatment, in our case plant diversity, and therefore with changes in vegetation, roofed controls do not necessarily have to be installed in every single replicate, With the use of such roofed controls it is possible to measure pure drought effects, but still under microclimatic conditions, which do not necessarily mimic future climate.

## Supporting Information

Figure S1 **In the first years of the Jena drought experiment we combined the diversity and drought treatment with an additional management treatment** [**20**] **using further roofs of the same construction reported here but with a different roof orientation.** Analysis of the extended dataset (including the additional random factor of revealed significant effects on PAR of the roof treatment (F_2,8.8_ = 12.42, p = 0.003), time of day (F_1,23.0_ = 298.46, p<0.001) as well as the interaction of the roof treatment and time (F_2,281.6_ = 18.88, p<0.001). Roofs reduced PAR by around 16% (corrected mean). The analysis of the reduced dataset (time span between 11 am and 3 pm) revealed only an effect of the roof treatment (F_2,85.4_ = 34.15, p<0.001), not of the time, indicating that the effect on PAR over the whole day was mainly determined by the difference between noon and the rest of the day. During noon roofs reduced PAR by ∼24%.
(TIF)

Table S1 **Nested design and statistical model specification for all response variables used in this study.** Nested structure gives information on the number of plots were used, whether they were nested in blocks and whether data were time series. All variables and contrasts used in the fixed term and the structure for the random term of the mixed effects models are listed.
(DOCX)

Table S2 **Fold changes (mean log response ratio, logRR) in metabolite levels due to pure drought (drought/roofed control) and roof artifacts (roofed control/ambient).** Only metabolites with significant differences due to either drought or roof artifact effects obtained by mixed effects models are presented. Significance of results is indicated by bold lettering.
(DOCX)
