# Supplementary figures and images for: Separating Drought Effects from Roof Artifacts on Ecosystem Processes in a Grassland Drought Experiment
Source: PLoS One. 2013 Aug 1;8(8):e70997. doi: 10.1371/journal.pone.0070997 (PMC3731277; doi:10.1371/journal.pone.0070997)

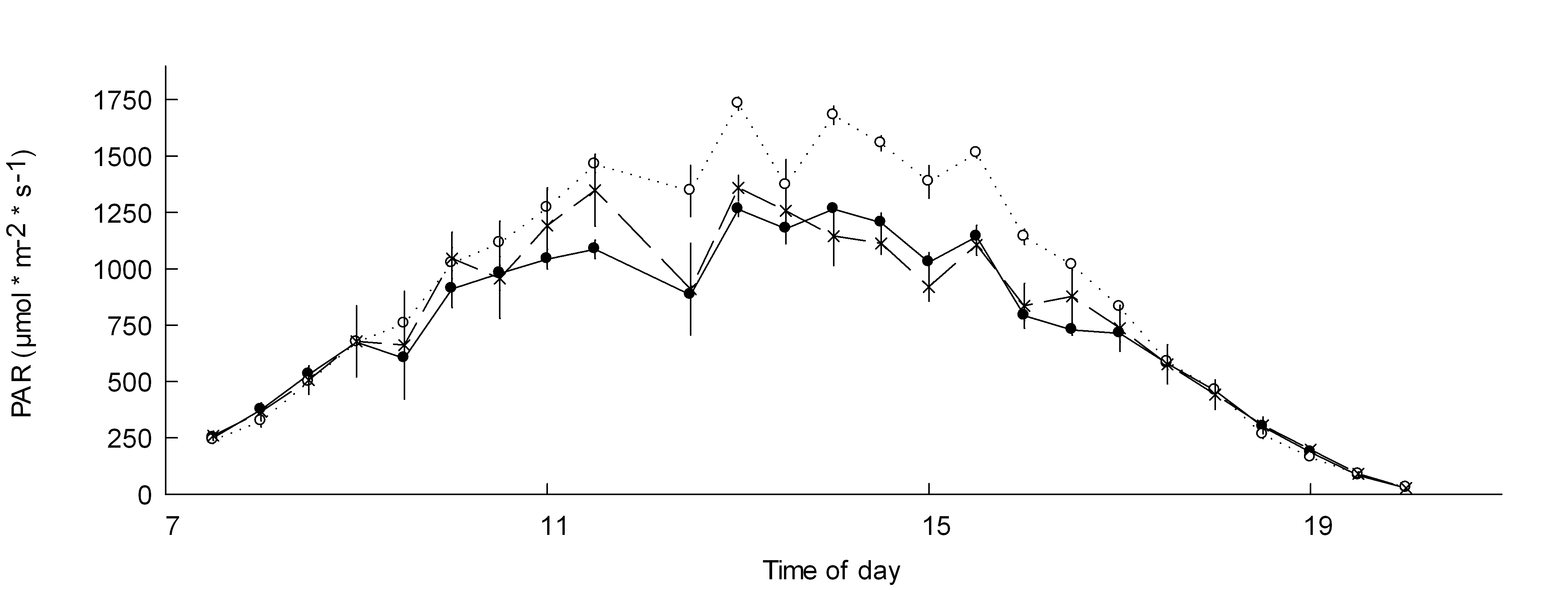

Supplement: Figure S1 — In the first years of the Jena drought experiment we combined the diversity and drought treatment with an additional management treatment [20] using further roofs of the same construction reported here but with a different roof orientation. Analysis of the extended dataset (including the additional random factor of revealed significant effects on PAR of the roof treatment (F2,8.8 = 12.42, p = 0.003), time of day (F1,23.0 = 298.46, p<0.001) as well as the interaction of the roof treatment and time (F2,281.6 = 18.88, p<0.001). Roofs reduced PAR by around 16% (corrected mean). The analysis of the reduced dataset (time span between 11 am and 3 pm) revealed only an effect of the roof treatment (F2,85.4 = 34.15, p<0.001), not of the time, indicating that the effect on PAR over the whole day was mainly determined by the difference between noon and the rest of the day. During noon roofs reduced PAR by ∼24%. (TIF) [file pone.0070997.s001.tif]
