# Supplementary material for: Separating Drought Effects from Roof Artifacts on Ecosystem Processes in a Grassland Drought Experiment
Source: PLoS One. 2013 Aug 1;8(8):e70997. doi: 10.1371/journal.pone.0070997 (PMC3731277; doi:10.1371/journal.pone.0070997)
Supplement: Table S1 — Nested design and statistical model specification for all response variables used in this study. Nested structure gives information on the number of plots were used, whether they were nested in blocks and whether data were time series. All variables and contrasts used in the fixed term and the structure for the random term of the mixed effects models are listed. (DOCX) [file pone.0070997.s002.docx]

**Table S1** Nested design and statistical model specification for all response variables used in this study. Nested structure gives information on the number of plots were used, whether they were nested in blocks and whether data were time series. All variables and contrasts used in the fixed term and the structure for the random term of the mixed effects models are listed.

|  | Nested structure | | | statistical model specification | |
| --- | --- | --- | --- | --- | --- |
| variable | Nr. Blocks | Nr. Plots_total_ | temporal | fixed term | random term |
|  |  |  |  |  |  |
| soil moisture | 3 | 3 | date | DvsRU+RvsU+date+DvsRU x date+RvsU x date | plot/subplot/date |
| soil temperature | 4 | 4 | date | WarmvsCold+DRvsU+DvsR+WarmvsCold x DRvsU+WarmvsCold x DvsR | plot/subplot/date+plot/subplot/WarmvsCold |
| air temperature | 4 | 4 | date | WarmvsCold+DRvsU+DvsR+WarmvsCold x DRvsU+WarmvsCold x DvsR | plot/subplot/date+plot/subplot/WarmvsCold |
| PAR | 1 | 4 | time | treatment+sinustime+treatment x sinustime | plot/subplot/sinustime+plot/subplot/time |
| aboveground biomass | 4 | 80 | - | Model A) SR_linear_(+leg)+DRvsU+DvsR+SR_linear_ x DRvsU+SR_linear_ x DvsR | block/plot/subplot |
|  |  |  |  | Model B) SR_linear_(+leg)+DvsRU+RvsU+SR_linear_ x DvsRU+SR_linear_ x RvsU | block/plot/subplot |
| litter decomposition | 4 | 80 | - | Model A) SR_linear_+DRvsU+DvsR+SR_linear_ x DRvsA+SR_linear_ x DvsR | block/plot/subplot |
|  |  |  |  | Model B) SR_linear_+DvsRU+RvsU+SR_linear_ x DvsRA+SR_linear_ x RvsU | block/plot/subplot |
| metabolites | 3 | 8 | - | Model A) DRvsU+DvsR | plot/subplot |
|  |  |  |  | Model B) DvsRU+RvsU | plot/subplot |

SR_linear_ = log-transformed variable of sown species richness tests for a linear relationship
