# Supplementary material for: Separating Drought Effects from Roof Artifacts on Ecosystem Processes in a Grassland Drought Experiment
Source: PLoS One. 2013 Aug 1;8(8):e70997. doi: 10.1371/journal.pone.0070997 (PMC3731277; doi:10.1371/journal.pone.0070997)
Supplement: Table S2 — Fold changes (mean log response ratio, logRR) in metabolite levels due to pure drought (drought/roofed control) and roof artifacts (roofed control/ambient). Only metabolites with significant differences due to either drought or roof artifact effects obtained by mixed effects models are presented. Significance of results is indicated by bold lettering. (DOCX) [file pone.0070997.s003.docx]

**Table S2** Fold changes (mean log response ratio, logRR) in metabolite levels due to pure drought (drought/roofed control) and roof artifacts (roofed control/ambient). Only metabolites with significant differences due to either drought or roof artifact effects obtained by mixed effects models are presented. Significance of results is indicated by bold lettering.

|  |  |  |  |  | logRR | |
| --- | --- | --- | --- | --- | --- | --- |
| Organ | Retention-index | Analyte-ID^a^ | Number in Figure 5 | Metabolite name | drought/ roof control | roof control/ambient |
| flower | 1533 |  |  |  | **1.66** | -0.51 |
| flower | 1600 | A160001 |  |  | -0.01 | **0.13** |
| flower | 1604 |  |  |  | 0.05 | **0.15** |
| flower | 1671 | A168001 | 1 | Asparagine^c^ | 0.02 | **0.49** |
| flower | 1736 |  |  |  | -0.01 | **0.16** |
| flower | 1770 |  |  |  | -0.02 | **0.20** |
| flower | 1813 | A182007 |  |  | 0.03 | **0.07** |
| flower | 1819 | A183011 |  |  | **1.50** | -0.94 |
| flower | 1928 |  |  |  | 0.02 | **0.10** |
| flower | 2007 | A201001 |  | Saccharic acid | -0.02 | **0.22** |
| flower | 2040 | A204001 |  | Galactaric acid^b^ | -0.06 | **0.18** |
| flower | 2070 | A209001 | 5 | Allantoin | 0.66 | **1.00** |
| flower | 2177 | A217004 |  |  | 0.02 | **-0.21** |
| flower | 2512 | A254002 |  |  | -0.51 | **1.66** |
| flower | 3112 | A313005 |  |  | 1.14 | **-1.48** |
| flower | 3796 |  |  |  | **-1.75** | 0.94 |
| flower | 4054 |  |  |  | -0.02 | **-1.64** |
| sink leaf | 1201 |  |  |  | -0.20 | **0.23** |
| sink leaf | 1428 |  |  |  | **-1.27** | 0.44 |
| sink leaf | 1598 | A161001 |  | Asparagine^c^ | **0.11** | -0.07 |
| sink leaf | 1720 | A171012 | 3 | Arabitol^b^ | 0.06 | **0.21** |
| sink leaf | 1770 |  |  |  | **0.79** | **-0.85** |
| sink leaf | 1943 | A195002 |  | Ascorbic acid | -0.07 | **-1.18** |
| sink leaf | 2134 |  |  |  | 0.36 | **-0.90** |
| sink leaf | 2177 | A217004 |  |  | -0.01 | **0.17** |
| sink leaf | 2308 | A233002 | 19 | Glucose-6-phosphate | 0.37 | **-1.87** |
| sink leaf | 2504 |  |  |  | 0.02 | **1.34** |
| sink leaf | 2595 | A259001 |  |  | 0.01 | **0.10** |
| sink leaf | 3087 |  |  |  | -0.46 | **2.15** |
| sink leaf | 3122 | A313007 |  |  | **0.12** | -0.09 |
| sink leaf | 3190 |  |  |  | 0.43 | **-2.02** |
| sink leaf | 3601 |  |  |  | **1.71** | 0.42 |
| source leaf | 1060 |  |  |  | **0.33** | -0.09 |
| source leaf | 1280 | A130002 |  |  | **0.24** | -0.03 |
| source leaf | 1378 | A140005 |  | Threonic acid-1.4-lactone^b^ | **0.08** | -0.02 |
| source leaf | 1551 | A155004 |  |  | **0.17** | **-0.19** |
| source leaf | 1600 | A160001 |  |  | **0.14** | 0.00 |
| source leaf | 1679 | A168002 |  | Ribose^b^ | **0.12** | -0.08 |
| source leaf | 1698 | A171001 | 15 | Xylitol^b^ | **0.13** | -0.04 |
| source leaf | 1739 | A174001 |  |  | **0.11** | -0.03 |
| source leaf | 1813 | A182007 |  |  | **0.31** | -0.12 |
| source leaf | 1840 |  |  |  | 0.09 | **-0.11** |
| source leaf | 1992 | A200001 |  | Gluconic acid^b^ | **0.10** | -0.01 |
| source leaf | 2011 | A201002 |  |  | **0.15** | -0.02 |
| source leaf | 2065 | A207006 |  |  | **0.18** | **-0.23** |
| source leaf | 2615 | A262001 |  |  | 0.80 | **-0.89** |
| **sum of significant changes (p<0.05)** | | |  |  | **20** | **29** |

^a^From the Golm metabolome database. ^b^Retention time index and fragmentation pattern were not sufficient to differentiate between closely related isomers. ^c^This compound was measured in the form of several analytes.
